# Decoding Lifespan Changes of the Human Brain Using Resting-State Functional Connectivity MRI

**DOI:** 10.1371/journal.pone.0044530

**Published:** 2012-08-30

**Authors:** Lubin Wang, Longfei Su, Hui Shen, Dewen Hu

**Affiliations:** College of Mechatronics and Automation, National University of Defense Technology, Changsha, Hunan, China; Hangzhou Normal University, China

## Abstract

The development of large-scale functional brain networks is a complex, lifelong process that can be investigated using resting-state functional connectivity MRI (rs-fcMRI). In this study, we aimed to decode the developmental dynamics of the whole-brain functional network in seven decades (8–79 years) of the human lifespan. We first used parametric curve fitting to examine linear and nonlinear age effect on the resting human brain, and then combined manifold learning and support vector machine methods to predict individuals' “brain ages” from rs-fcMRI data. We found that age-related changes in interregional functional connectivity exhibited spatially and temporally specific patterns. During brain development from childhood to senescence, functional connections tended to linearly increase in the emotion system and decrease in the sensorimotor system; while quadratic trajectories were observed in functional connections related to higher-order cognitive functions. The complex patterns of age effect on the whole-brain functional network could be effectively represented by a low-dimensional, nonlinear manifold embedded in the functional connectivity space, which uncovered the inherent structure of brain maturation and aging. Regression of manifold coordinates with age further showed that the manifold representation extracted sufficient information from rs-fcMRI data to make prediction about individual brains' functional development levels. Our study not only gives insights into the neural substrates that underlie behavioral and cognitive changes over age, but also provides a possible way to quantitatively describe the typical and atypical developmental progression of human brain function using rs-fcMRI.

## Introduction

Resting-state functional connectivity MRI (rs-fcMRI) has emerged as a powerful tool for investigating functional brain organization. This method is based on the discovery that functionally related brain areas have correlated spontaneous low-frequency (<0.1 Hz) blood oxygen level-dependent (BOLD) signal fluctuations [1], [2], and has been used to explore brain networks involved in motor [3], language [4], sensory [5], memory [6], [7], attention [8], and reading [9] systems. Age-related changes of resting-state functional networks may provide neural substrates that underlie the maturation of behavioral and cognitive functions, and the inevitable functional decline in advanced aging. Furthermore, better understanding the developmental dynamics of normal brain organization is urgently needed for early diagnosis and therapy of developmental neuropsychiatric disorders such as Alzheimer's disease and autism.

Increasing developmental rs-fcMRI studies have shown significant age effect on the organization of functional brain networks. For example, over maturation, the organization of multiple functional networks shifts from a local anatomical emphasis in children to a more distributed architecture in young adults, by the weakening of short-range functional connections and the strengthening of long-range functional connections [10]–[14]. It has also been demonstrated that normal aging is associated with functional segregation of large-scale brain systems that support higher-level cognition [15]–[17]. To date, the majority of research efforts are focused on investigating functional brain development within a limited age range. However, the developmental dynamics of the resting human brain across the lifespan have not yet been well studied. Moreover, the spatial and temporal patterns of age effect on resting-state functional networks remain unclear. Previous structural brain imaging studies have found regional differences in brain maturation and aging [18]–[21]. A recent rs-fcMRI study has also observed regionally specific age-related changes in functional homotopy [22]. It is probable that functional connectivity in different brain systems exhibit specific developmental trajectories of varying levels of complexity.

Based on previous findings of reliable changes in resting-state functional networks over age, an emerging field is to predict single individuals' “brain ages” from rs-fcMRI data, which is potentially useful to aid in the diagnosis of individuals with disordered brain function. The advantage of resting-state neuroimaging includes that it is easy to acquire without any complicated task design, and thus can be readily accepted by participants who lack the ability to perform task, such as children and patients. Recently, Dosenbach et al. [23] used rs-fcMRI to make predictions about brain maturity of typically developing individuals (ages 7 to 30 years). However, due to the complex patterns of age-related changes in functional brain networks from childhood into senescence, it is more challenging to learn a predictable model of functional brain development across the human lifespan. A possible way to resolve this issue is to find compact representations that capture the intrinsic dimensions of brain maturation and aging. Similar to the manifold way of face image analysis for age estimation [24], [25], we hypothesized that the developmental dynamics of human brain function reside on a low-dimensional manifold embedded in the high-dimensional functional connectivity space. From this point of view, we expected that powerful manifold learning algorithms could uncover the inherent structure of age-related changes in resting-state functional networks that affords physiological interpretability.

In this study, we investigated the developmental dynamics of the resting human brain encoded in the whole-brain functional network of 137 normal subjects ranging in age from 8 to 79 years. The BOLD time series were extracted from 116 brain regions according to the anatomically labeled (AAL) atlas [26]. All possible interregional functional connections were computed to create the whole-brain functional network. We first examined linear and nonlinear age effect on each functional connection using parametric curve fitting. For functional connections exhibiting nonlinear developmental trajectories, the ages associated with maximum or minimum connectivity strength were also examined to study at what age the brain stop “maturing” and start “aging”. We then proposed a pattern regression framework to predict individuals' “brain ages” from the whole-brain functional network. In the framework, a supervised Locality Preserving Projections (LPP) algorithm [27] was employed to learn a low-dimensional representation of brain development from many individuals at different ages; and support vector regression (SVR) models were designed in the manifold coordinate space for making continuously valued predictions about the functional development levels of individual brains.

## Methods

### Subjects and imaging protocols

Two public resting-state fMRI datasets with broad age range were selected from the freely accessible image repository for the 1000 Functional Connectomes Project (http://www.nitrc.org/projects/fcon_1000/) [2]. These data were scanned at two centers: New York University (NYU) and International Consortium for Brain Mapping (ICBM). Freely publishing any portion of the data in the web-based repository is approved by the 1000 Functional Connectomes Project.

At NYU, 84 normal subjects (age range 8–49 years; 43 males) underwent resting-state scans on a 3-T MRI scanner with the following parameters: 192 time points; 39 axial slices; repetition time  = 2000 ms; voxel size  = 3×3×3 mm^3^; slice acquisition order  =  interleaved ascending. At ICBM, 86 normal subjects were collected for brain imaging on a 3-T MRI scanner. Only the 53 subjects (age range 19–79 years; 22 males) with full brain coverage scanning were included in the present analyses, each of which had two resting-state scans. The imaging parameters were as follows: 128 time points; 23 axial slices; repetition time  = 2000 ms; voxel size  = 4×4×5.5 mm^3^; slice acquisition order  =  sequential descending. Characteristics of different age groups in this study are shown in Table 1.

**Table 1:** Characteristics of different age groups in this study.

Age group	Number of subjects	Gender (M/F)	Handedness (L/R)	Head motion in mm (mean ± sd)
8–20	34	18/16	2/32	0.194±0.123
21–30	50	22/28	1/49	0.121±0.048
31–40	11	5/6	0/11	0.160±0.060
41–50	16	7/9	0/16	0.173±0.067
51–60	7	5/2	0/7	0.199±0.045
61–70	10	5/5	0/10	0.247±0.129
71+	4	1/3	0/3^a^	0.288±0.053

a in the 71+ group, the handedness of one subject is ambidextrous.

### Data preprocessing

The first five functional images of each scan have already been discarded in the “1000 Functional Connectomes Project” release, to remove possible T1 stabilization effects. All resting-state images were preprocessed using the statistical parametric mapping software package (SPM5, Wellcome Department of Cognitive Neurology, Institute of Neurology, London, UK). The data were corrected for within-scan acquisition time differences between slices, and realigned to the first volume to correct for inter-scan head motions. Six realignment parameters [*d_x_*, *d_y_*, *d_z_*, *α*, *β*, *γ*] were obtained from the rigid body correction of head motion (translational and rotational displacements along *x*, *y*, and *z* axes). For the *i*-th time point, we used framewise displacement to represent instantaneous head motion, which is defined by [28]:
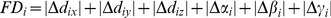
(1)where 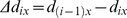, and similarly for the other realignment parameters. Rotational displacements were converted from degrees to millimeters by calculating displacement on the surface of a sphere of radius 50 mm, which is approximately the mean distance from the cerebral cortex to the center of the head. For each subject, the framewise displacements were averaged across time points to express his/her mean head motion during scanning. In this study, the mean head motion of every subject was less than 1 mm. Even though head motion was small, it varied among different age groups (Table 1). In general, children and old adults had larger head motion than young adults.

The volumes were normalized to the standard EPI template in the Montreal Neurological Institute (MNI) space and resliced to 3×3×3 mm^3^. Then the data were spatially smoothed with a Gaussian filter of 8 mm full-width half-maximum kernel. The smoothed images were temporally band-pass filtered (0.01–0.1 Hz), followed by linear detrending to remove any residual drift. Nine nuisance signals were removed from the time series of each voxel via linear regression, including white matter (WM) signal, cerebrospinal fluid (CSF) signal, the whole-brain signal, and six motion parameters. The whole-brain signal was generated by averaging across the times series of all voxels in the brain. The WM and CSF signals were generated by averaging across the times series of a region centered in the white matter and a ventricular region of interest, separately. The six motion parameters were obtained by rigid body correction of head motion. This regression procedure was utilized to reduce spurious variance unlikely to reflect neural activity.

The fMRI volumes registered with the MNI template were further divided into 116 gray matter regions according to the AAL atlas [26]. This template parcellates the cerebrum into 90 regions (45 in each hemisphere) and the cerebellum into 26 regions (9 in each cerebellar hemisphere and 8 in the vermis). All region of interest (ROI) masks were generated by using the software WFU_PickAtlas (http://www.ansir.wfubmc.edu). Regional mean time series were extracted by averaging the fMRI time series over all voxels in each of the 116 regions. The mean time series from each region was then correlated with the time series from all other regions using Pearson's correlation coefficient, creating a whole-brain functional network captured by a 116×116 symmetric matrix. Fisher's z-transform was applied to the correlation values to ensure normality. Finally, the upper triangle elements of the functional connectivity matrix were extracted as features in all subsequent analyses.

### Parametric curve fitting

Following previous studies of brain development [19], [22], we used the following two models to fit linear and quadratic functional connectivity changes over age.
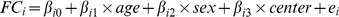
(2)

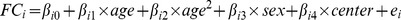
(3)where 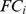 denotes the *i*-th interregional functional connection, and 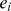 denotes the random error. Both sex and center factors were modeled as 0–1 covariates (e.g., 0: male; 1: female; 0: the NYU center; 1: the ICBM center). Akaike's information criterion [29], [30] was used to select the best-fit model, which reflects a trade-off between the likelihood and complexity of a model.

For each functional connection, the T statistic for coefficients of *age* in Equation (1) was used to measure the significance of linear developmental trajectories, and the T statistic for coefficients of *age^2^* in Equation (2) was used to measure the significance of quadratic developmental trajectories once the linear effects of age had been removed. Significance level was set at *p*<0.0001. The underlying mechanisms of negative functional connectivity are still under debate, which can correspond to a state of anticorrelation between brain regions [31], or be introduced by whole-brain signal regression [32]. In this study, we hence focused on functional connections exhibiting positive values across subjects (one sample t-test, *p*<0.001). To obtain robust statistical results, the parametric curve fitting was performed *N* times (*N* is the number of available subjects), each time leaving one subject out. Functional connections were selected as significant only if they satisfied the above two criterions for each time of the analyses. For functional connections exhibiting quadratic developmental trajectories, the ages associated with maximum or minimum connectivity strength were calculated from the first derivatives of the fitted quadratic curves, to study at what age the brain stop “maturing” and start “aging”.

### Age prediction framework

**Figure 1:**
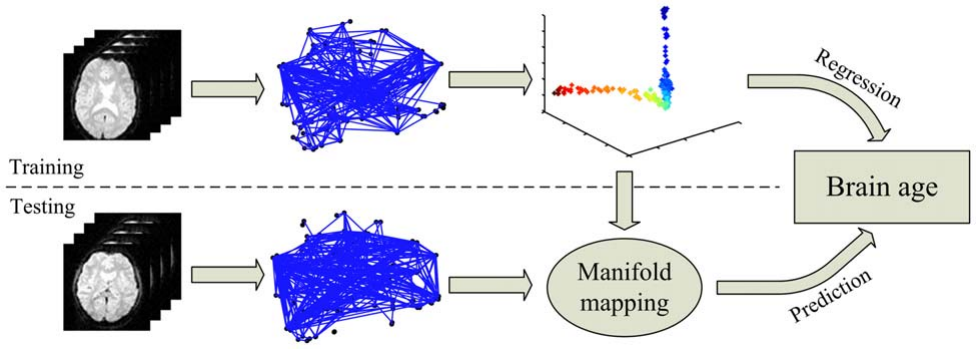
Flow chart of age prediction via rs-fcMRI.

In this study, the whole-brain functional connectivity pattern was used as the feature for age prediction. Our age prediction framework consisted of two steps. In the training step, the LPP algorithm was used to find a low-dimensional manifold that represented the lifespan dynamic process of the whole-brain functional network; a regression model was then designed to characterize the relationship between manifold coordinates and age. In the test phase, the low-dimensional embeddings of new test samples were extracted and fitted with the learned regression model to make prediction of their “brain ages”. The flow chart of age prediction via rs-fcMRI is shown in Figure 1.

We hypothesized that the lifespan developmental dynamics of the resting human brain reside on or close to a smooth low-dimensional manifold embedded in the functional connectivity space. Suppose the high-dimensional connectivity space consists of *N* data points 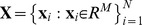 with dimensionality *M*. 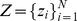 associated with the data points provides the corresponding subjects' chronological age labels. Our goal was to find a low-dimensional discriminative manifold embedded in the functional connectivity space and a low-dimensional representation 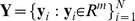 with 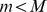. To easily generalize the embeddings to new data points, we chose the LPP algorithm to learn a manifold associated with functional brain development across the human lifespan. The manifold preserved characterization of local geometric structure in the functional connectivity space by nearest-neighbor graph. The algorithmic procedure of LPP is formally stated in Text S1 and the theoretical foundation of LPP is detailed in He et al. [27]. We incorporated the age labels into the embedding process in a supervised manner. The basis idea is to multiply a penalty term 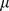 on the Euclidean distance between 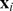 and 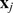 if the age gap of the *i*-th and *j*-th subjects was larger than 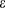, which was defined as follows:
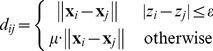
(4)


To validate the effectiveness of LPP, a classical dimensionality reduction algorithm, Principle Component Analysis (PCA), was also used to find a low-dimensional embedding of the rs-fcMRI data, which projects the data along the directions of maximal variance.

Support vector machine belongs to a learning system based on recent advances in statistical learning theory, and is widely used in computational biology [33]. By introducing a loss function, support vector machine can be applied to regression problems. As in Dosenbach et al. [23], chronological age served as the measure for brain development prediction, which is easily obtained and free of measurement error. In this study, support vector regression (SVR) was employed to characterize the relationship between the embedded features, 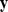, and the age labels, *Z*. Based on these regression models, the “brain ages” of new subjects can be predicted as an estimate of their functional development levels.

Using a quadratic loss function, SVR determines the parameter vector, 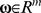, and bias, 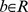, of a linear function, 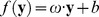, via minimizing the following regularized risk function:
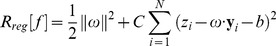
(5)


The term 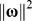 is characterized as model complexity; *C* is a constant to trade off the empirical risk and model complexity. In this study, the parameter *C* was selected from the range of 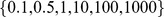. Regression models can be made nonlinear by using a kernel trick, which entails mapping the data points to a higher dimensional feature space and applying a linear function in this space. Though the regression function is linear in the higher dimensional feature space, it is nonlinear in the input space. In this study, the Gaussian radial basis function kernel was used for nonlinear SVR. A radial basis function is of the form:
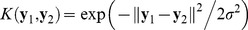
(6)where 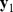 and 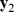 are two data points in the input space, and 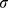 adjusts the width of the Gaussian kernel. We selected 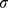 from the range of 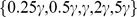, where 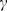 was the average distance from all pairs of training data points.

Assuming that brain development in a small age range was close to linear, multiple piecewise linear functions can be used to approximate the nonlinear age-related changes in the brain. In this study, we proposed a locally adjusted SVR (LASVR) method for local adjustment of the global regression results. For a new sample 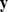, suppose the estimated age value by linear SVR is 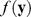. The idea of the LASVR method is to learn a local model 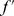 using training samples within a limited range of ages centered at the global regression result, i.e., 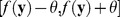, and adjust the initially estimated age value 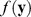 to 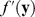. The parameter 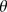 is a predefined parameter indicating the range of ages for local adjustment. Therefore, the LASVR method is a multistage regression scheme with four steps: (1) learn a global linear SVR model based on all the training samples; (2) make prediction about a new sample using the global linear SVR; (3) learn a local linear SVR model based on training samples within a limited range of ages centered at the global regression result; (4) adjustment of the estimated age of the new sample using the local regression model.

Because of the limited number of samples, leave-one-out cross-validation (LOOCV) was conducted to estimate the prediction accuracy of our method. In each LOOCV round, one sample was designated the test sample while the remaining ones were used as the training samples. Note that subjects collected by ICBM scanned twice, so we performed LOOCV across subjects, not scans; and the estimated age for each subject in ICBM was an average of the predictions from the two scans of this subject. The prediction performance was quantified by the mean absolute error (MAE) and cumulative score (CS) on the basis of LOOCV results. MAE was defined as the average of the absolute errors between the estimated ages and chronological ages:
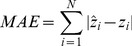
(7)where 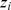 is the chronological age for sample *i*, 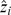 is the estimated age, and *N* is the total number of subjects. The cumulative scores were defined as the percent of samples correctly predicted at different error levels. Let 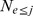 be the number of samples on which the age prediction makes an absolute error no higher than *j* years, the cumulative score at error level *j* was calculated by:
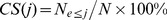
(8)


## Results

### Age-related changes in interregional functional connectivity

**Figure 2:**
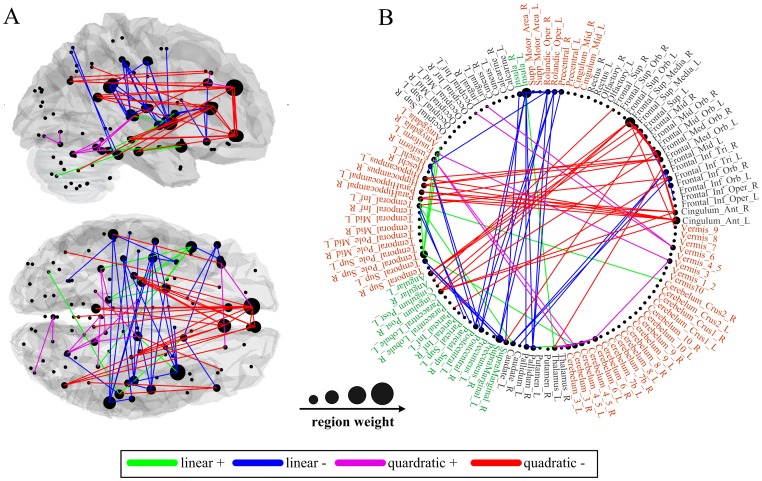
Age-related changes in interregional functional connectivity displayed on (A) a surface rendering of the brain and (B) a schematic diagram. Connections with positive linear, negative linear, positive quadratic and negative quadratic developmental trajectories are shown in green, blue, magenta, and red, respectively. Also displayed are the brain regions scaled by their weights (sum of the T statistics for all the connections passing through that brain region).

**Figure 3:**
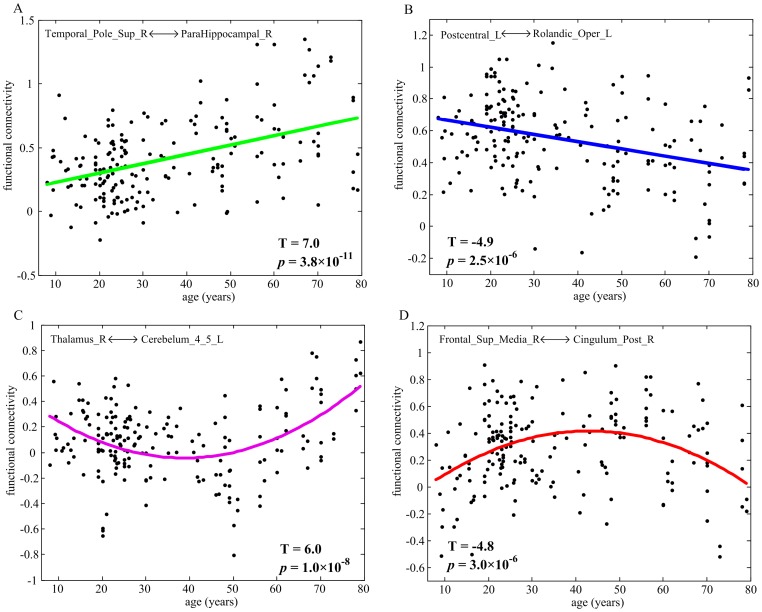
The typically developmental trajectories of resting-state functional connectivity. (A) positive linear change; (B) negative linear change; (C) positive quadratic change; (D) negative quadratic change.

A summary of functional connections that exhibited significant linear or quadratic changes over age (*p*<0.0001) are graphed in Figure 2. The four types of typically developmental trajectories are also shown in Figure 3. Linear decreases with age were mainly found in functional connectivity between central parts of the brain, comprising bilateral regions of motor, somatosensory, temporal and parietal association cortex, and insula, as well as several prefronto-basal ganglia connections (Figure 2, blue connections). In contrast, linear increases with age were found in functional connectivity of brain regions related to the affective function, such as the superior temporal pole [34], amygdala [35], parahippocampal gyrus [36], and fusiform gyrus [37] (Figure 2, green connections).

Many prefronto-temporal and prefronto-parietal connections exhibited negative quadratic developmental trajectories (Figure 2, red connections). These connections showed age-related increases during childhood and early stage of adulthood, whereas decreases later in life (inverted U-shaped). In contrast, positive quadratic developmental trajectories were found in functional connections between brain regions close in anatomical space, such as connections within the cerebellum and prefrontal cortex, and connections between cerebellum and posterior parts of the cortex (Figure 2, magenta connections). These connections exhibited age-related decreases during childhood and early stage of adulthood, whereas increases later in life (U-shaped). The mean peak ages for connections exhibiting positive and negative quadratic trajectories were 38 and 40 years, respectively. Moreover, we did not observe significant variation of these two types of peak ages across functional connections (positive range of 34–45 years, SD of 3.2; negative range of 34–47 years, SD of 2.9).

**Figure 4:**
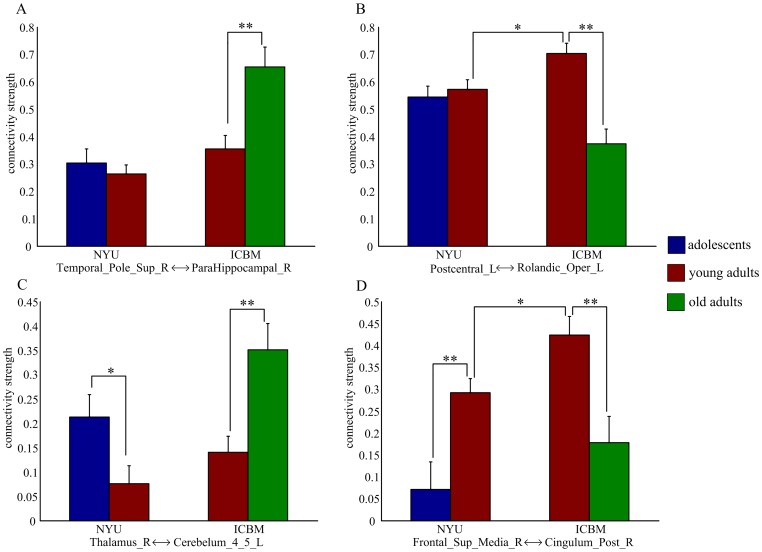
Group- and center-related differences for the developmental trajectories of resting-state functional connectivity shown in **Figure 3**. (A) positive linear change; (B) negative linear change; (C) positive quadratic change; (D) negative quadratic change. The error bars represent standard error of mean. *: *p* <0.05, **: *p* <0.005, two-sample t-test.

The developmental trajectories of resting-state functional connectivity (Figure 3) were further examined in the NYU data and ICBM data, respectively. Adolescents (ages 8–15 years) and young adults (ages 21–30 years) were selected from the NYU data, and young adults (ages 21–30 years) and old adults (ages 61–79 years) were selected from the ICBM data. Though there were center-related variations in the strength of functional connectivity, reliable age effect remained appreciable in each single center (Figure 4). For the ICBM data, significant group differences between young adults and old adults were observed in both linear and nonlinear developmental trajectories of functional connectivity. For the NYU data, significant group differences between adolescents and young adults were observed only in the nonlinear developmental trajectories, suggesting that the linear developmental trajectories mainly represented the aging process.

### Low-dimensional embeddings

**Figure 5:**
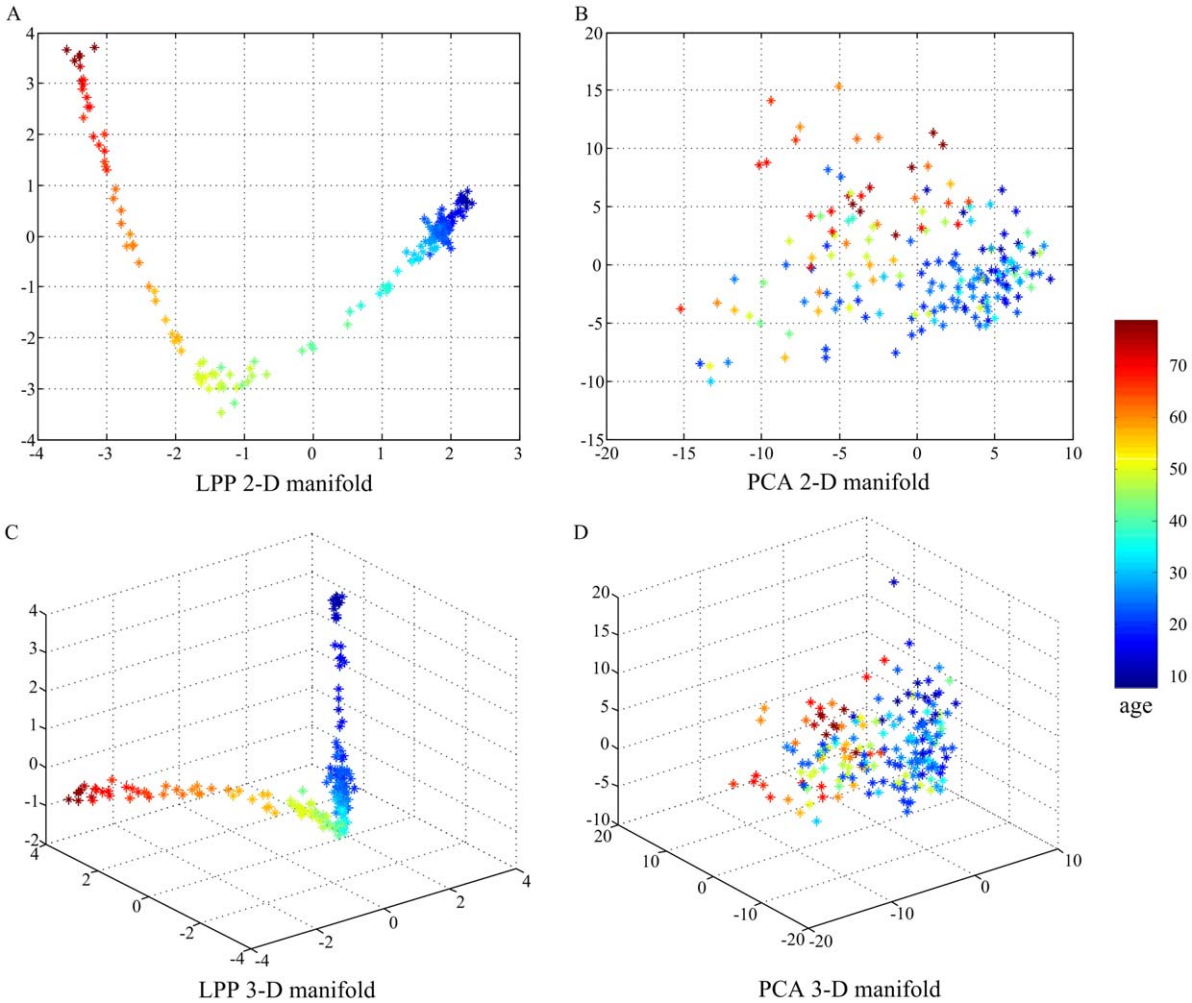
Low-dimensional embeddings of age-related changes in the whole-brain functional network. The first 2-D and 3-D embeddings learned by LPP are plotted in (A) and (C). The first 2-D and 3-D embeddings learned by PCA are plotted in (B) and (D). Each data point represents one subject. The data points of age from 8 to 79 years are colored from blue to red.

Figure 5 shows the 2-D and 3-D embeddings of age-related changes in the whole-brain functional network (6,670 functional connections) based on the LPP and PCA algorithms. The embedding achieved by LPP approximately formed a nonlinear curve with subjects distributing on it in the chronological way. Moreover, the typically maturational and aging processes of the resting human brain resided on two “branches” of the curve. The intersection of the two “branches” was in the fourth decade, which was consistent with the mean peak age of functional connections exhibiting quadratic developmental trajectories. In contrast, PCA produced a mapping with no clear manifold trend or structure and failed to capture the nonlinear structure of age-related changes in the resting human brain in a low-dimensional linear subspace. A parameter, the number of nearest neighbors *k*, was set to be 8 in LPP algorithm. Some different values of *k* were also tested (Figure S1). We found that the low-dimensional structure obtained by LPP was relative stable to *k*. However, a too small *k* could falsely divide the continuous developmental progression into disjointed sub-manifolds. In contrast, if *k* was too large, important structures of the manifold were smoothed or eliminated, causing that the positions of children and old subjects were adjacent on the manifold. It should be pointed out that the low-dimensional embedding coordinates might be changed when the LPP algorithm runs at another time, but the geometric structure holds all along.

### Prediction performance

**Figure 6:**
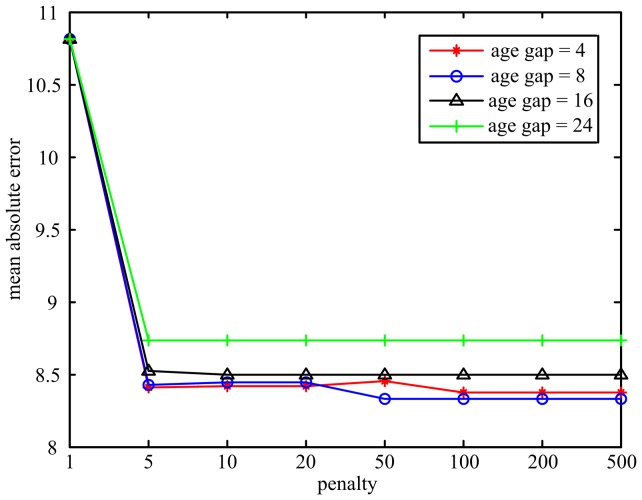
The mean absolute error curves under different age gap and penalty parameters in LPP.

Two parameters (age gap and penalty) should be set in the supervised LPP algorithm. In our age prediction framework, we first evaluated how the age gap and penalty parameters in LPP impact the prediction performance. Linear SVR was chosen as the regression model (*C* was fixed to 0.1). The mean absolute error curves under different age gap and penalty parameters are plotted in Figure 6. We found that increasing the penalty parameter could sharply reduce the mean absolute error. When the penalty was sufficient large (>100), the prediction performance did not change. On the other hand, a too large age gap would potentially reduce the supervisory ability of LPP, and resulted in lower prediction performance. In the following analyses, we set age gap to be 8 and penalty to be 100.

Several SVR algorithms were further employed to model the manifold coordinates with age. Table 2 shows the best prediction results of different SVR algorithms with respect to the number of reduced dimensions. Using linear SVR, the mean absolute error between the predicted “brain ages” and chronological ages was 8.3 years, and there were about 67% and 93% test samples with an absolute error no higher than 10 years and 20 years, respectively. The performance of nonlinear SVR was slightly higher than that of linear SVR, with a mean absolute error of 8.2 years. Different local adjustment ranges, e.g. 4, 8, and 16, were tried for LASVR. When the adjustment range was set to be 8, the mean absolute error was reduced to about 7.5 years, and the percent of test samples with an absolute error no higher than 10 years was increased to about 75%. For all regression algorithms, the mean absolute error decreased as the dimensionality of the manifold increased to a particular number. There was no significant improvement of prediction performance if more dimensions were used, suggesting that age-related changes in the whole-brain functional network can be parameterized by a small number of variables.

**Table 2:** Comparison of prediction performance using different SVR algorithms.

Methods	dimension	MAE	CS(5)	CS (10)	CS(20)
linear SVR	7	8.3	39%	67%	93%
nonlinear SVR	4	8.2	40%	69%	94%
LASVR (4)	13	7.8	43%	73%	93%
LASVR (8)	7	7.5	45%	75%	92%
LASVR (16)	4	7.7	46%	72%	93%

MAE denotes the mean absolute error between the predicted ages and chronological ages. CS(*j*) denotes the percent of test samples with an absolute error no higher than *j* years. Different local adjustment ranges (4, 8, and 16) were tried for LASVR.

## Discussions

In this study, we used machine learning methods to decode the developmental dynamics of the whole-brain functional network in seven decades (8–79 years) of the human lifespan. We observed significant age-related changes in interregional functional connectivity with spatially and temporally specific patterns. During brain development from childhood to senescence, functional connections tended to linearly increase in the emotion system and decrease in the sensorimotor system; while quadratic trajectories were observed in functional connections related to higher-order cognitive functions. We further demonstrated that the complex age effect on the whole-brain functional network could be effectively represented by a low-dimensional, nonlinear manifold, which uncovered the inherent structure of brain maturation and aging. Regression of the manifold coordinates with age showed that the manifold representation extracted sufficient information from rs-fcMRI data to make prediction about individual brains' functional development levels.

### Spatially and temporally specific development of the whole-brain functional network

Linear decreases with age were mainly found in functional connections between central parts of the brain, comprising bilateral regions of motor, somatosensory, temporal and parietal association cortex, and insula. In addition, some prefronto-basal ganglia connections were also linearly decreased with age. It is consistent with recent rs-fcMRI evidence that the sensorimotor system may be disrupted in aging [17], [38]. Compared with young adults, old subjects exhibit a great extent of activation in the sensorimotor cortex, as well as the recruitment of additional areas (particularly the prefrontal and basal ganglia regions) for successful motor performance [39]–[41]. The functional connections between these brain regions are vulnerable to age-related effects, resulting in an imbalance of “supply and demand”. Based on previous studies elucidating relationships between age-related brain differences and sensorimotor deficits in old subjects [42], we conjecture that the disruptive alterations of functional connectivity within the sensorimotor system may be an important neural mechanism contributing to the deteriorated sensorimotor control and functioning in aged subjects.

Previous task-related neuroimaging studies have reported better affective well-being and emotional stability with increasing age, which is associated with an adaptive shift toward greater controlled processing of negative emotion, with less control for positive emotion [43], [44]. In this study, linear increases with age were found in functional connectivity of emotion-related brain regions, possibly reflecting increased emotional regulation. We also observed that many linearly increased functional connections were associated with the superior temporal pole, which plays an important role in coupling social and emotional responses to highly processed sensory stimuli [34]. For example, functional connectivity between the superior temporal pole and parahippocampal gyrus may be involved in emotion-mediated memory formation [36]. Besides, the fusiform gyrus combining with superior temporal pole may be involved in the perception of emotional facial expression [37]. It has been demonstrated that correlated spontaneous brain activity can reflect histories of coactivation between brain regions [45], [46]. Therefore, cumulative effect of experiences across the human lifespan may not only alter the activation pattern for emotional stimuli, but also strengthen resting-state functional connectivity within the emotion system.

Many prefronto-temporal and prefronto-parietal connections exhibited inverted U-shaped developmental trajectories. Such connections were found to be related to the brain's default mode network (DMN), which is involved in monitoring internal and external environment, memory, and self-referential thought [6], [7], [31], [47], [48]. In addition, some prefronto-parietal connections were related to the task-positive network (TPN), which is associated with top-down modulation of attention and working memory [8], [31]. Previous studies have shown that normal aging is accompanied by marked reductions of functional correlations within higher-order brain systems, and the reduced correlations are associated with poor cognitive performance across a range of domains [15], [16], [49]. Other reports on children have shown that brain regions related to cognitive functions are only sparsely connected at children; over development, these regions integrate into cohesive, interconnected networks [10], [11], [13]. These findings strongly support our result that the strength of anterior-posterior functional connectivity within the DMN and TPN increased from childhood to adulthood but subsequently declined in old age. Moreover, we found that the mean peak age for the inverted U-shaped developmental trajectories was in the fourth decade. Interestingly, a recent longitudinal study has found that cognitive decline is already evident in middle age from the examination of more than 7,000 individuals aged 45–70 years at baseline [50]. Therefore, the progressive integration and disruption of the DMN and TPN may actually participate in the improvement and deterioration of cognitive abilities across the human lifespan.

Functional connections with U-shaped developmental trajectories were found between regions close in anatomical space. Previous studies have shown that the maturation of functional brain organization is characterized by simultaneously weakening of short-range functional connections and strengthening of long-range functional connections [12], [14], [23]. It is, to some extent, consistent with our findings that there was no significant difference between the mean peak ages for the inverted U- and U-shaped functional connectivity changes. An intriguing possibility is that in children and old subjects, the lack of efficient communication between distant regions may be compensated for by facilitating communication between anatomically proximal regions. It should be noted that some short-range functional connections in the prefrontal cortex exhibited inverted U-shaped developmental trajectories (Figure 2). Fair et al. [12] proposed that these short-range connections likely contributed to the most efficient “solution” for general task completion and remain in use during the adulthood.

Age-related changes in resting-state functional connectivity may be originated from multiple sources of developmental change. Because increased brain activity can be partly explained by increased GM volume [51], [52], it is possible that some of the trends seen here are related to GM volume changes with age. For example, sensorimotor-related brain regions, including the pre- and postcentral gyri, and insula, have been found to exhibit significant age-related loss in GM volume [53], [54], which may be responsible for the disruptive alterations of functional connectivity within the sensorimotor system in normal aging. Previous diffusion tensor imaging studies have demonstrated that the timing of protracted growth in white matter microstructure is in the fourth decade [20], [21], [55]–[58], which is consistent with the mean peak age for the functional connections with inverted U-shaped developmental trajectories. Therefore, the lifespan development of functional connectivity within higher-order brain systems may relate, in part, to the development of myelinated fibers that connect neurons in different cortical regions. By the combination of PiB PET and fMRI imaging techniques, it is shown that amyloid deposition is associated with abnormal brain activity in the DMN in older adults without dementia [59], suggesting that amyloid deposition could also be a potential biophysical factor for functional disruption of higher-order brain systems.

### Manifold representation of lifespan developmental dynamics in the human brain

Some recent studies have shown that manifold learning can be used to extract exciting new information from high-dimensional neuroimaging data [60]–[62]. In this study, we used manifold learning to uncover the inherent structure of age-related changes in the whole-brain functional network. The manifold achieved by LPP approximately formed a nonlinear curve, and the typically maturational and aging processes resided on two “branches” of the curve. The intersection of the two “branches” was in the fourth decade, which was consistent with the mean peak age of functional connections exhibiting quadratic developmental trajectories. Moreover, we found that the best prediction result could be achieved using only a few number of manifold coordinates (Table 2). Therefore, it is reasonable to consider that the complex patterns of age effect on the whole-brain functional network could be effectively represented by a nonlinear manifold embedded in the high-dimensional functional connectivity space. These impressive results provide the promise that manifold learning may aid visualization of the developmental dynamics of human brain function in a low-dimensional space.

PCA failed to capture the inherent structure of brain development in a low-dimensional linear subspace. As we discussed before, age-related changes in the whole-brain functional network probably reside on a nonlinear manifold. However, PCA finds a low-dimensional embedding that preserves most of the variance of the data, and lacks of the ability to discover the underlying structure if the data points lie on a nonlinear manifold hidden in the high-dimensional space. LPP optimally preserves local neighborhood information of the data by finding the optimal linear approximations to the eigenfunctions of the Laplace Betrami operator on the manifold [27]. As a result, LPP shares many of the data representation properties of nonlinear techniques such as Laplacian Eigenmaps [63] or Locally Linear Embedding [64], and is capable of discovering the nonlinear structure of the manifold. Another important reason is that, the age information was incorporated into the LPP embedding process for manifold learning in a supervised manner, while the traditional PCA method only worked in an unsupervised manner in learning the subspace representation.

### Predicting individual brains' functional development levels from rs-fcMRI data

It is argued that a suitable machine learning method in neuroimaging data analysis not only provides accurate predictions by exploiting discriminative information encoded in “hidden” physiological quantities, but also enables an intuitive and mechanistic interpretation [65]. In this study, we addressed these two issues by incorporating discriminative compact representation of rs-fcMRI data into regression models. Our method made continuously valued predictions about individual brains' functional development levels with a mean absolute error of 7.5 years, and there were about 75% test samples on which the age estimation made an absolute error no higher than 10 years. Moreover, the low-dimensional structure associated with brain maturation and aging underlying spontaneous brain activity was successfully extracted.

There are two reasons for the disparity between the predicted “brain ages” and chronological ages. On the one hand, imprecise of the regression model could cause prediction errors. Because of nonlinear age-related changes of the human brain, linear SVR could not model individual brains' functional development levels accurately. However, we found that nonlinear SVR did not provide a significant advantage in performance. A possible explanation is that the number of training samples available in this study was too small to reflect complicated relationships between features, and nonlinear SVR would overfit the regression model [33], [66]. We further proposed an algorithm to locally adjust linear SVR results, which resulted in better prediction performance. On the other hand, due to interindividual variations in experience and gene, a systemic error will be introduced when using chronological age as the measure of brains' functional development level [46], [67]. Only future experiments can determine the magnitude of this systemic error; however, our primary results suggest that a general developmental pattern of human brain function can be learned from many individuals at different ages.

### Methodology consideration

Smoothing or not smoothing the fMRI data is under debate. In this study, we have included smoothing as a preprocessing step to improve the signal-to-noise ratio, by assuming that adjacent voxels may have some independent noise, but similar signals of interest. However, smoothing the data will potentially introduce artificial connections, especially for voxel-based connectome analysis. The bias smoothing introduces is not expected to have significant impact on large-scale (here AAL parcellation) connectome analyses. To test this, the same analysis was repeated with unsmoothed data. We found that the pattern of age-related changes in the whole-brain functional network obtained with unsmoothed data (Figure S2) was very similar to that obtained with smoothed data. Moreover, our results showed that smoothing had little impact on the prediction accuracy of individual brains' functional developmental levels (Table S1).

AAL atlas is a structural parcellation of the brain and would combine functionally distinct ROIs [68], [69]. We additionally performed the analysis using 160 functional ROIs derived from a series of meta-analyses of task-related fMRI studies [23], and found that the majority of our results were not altered (see Figure S3 and Table S2). There were also some disparities. For example, more functional connections were identified to exhibit significant age-related changes, suggesting that accurate functionally defined ROIs have increased sensitivity in measuring functional connectivity. However, it is not suitable to study age effect on the emotional system using the 160 functional ROIs, due to that the generation of these functional ROIs did not include emotional tasks. Therefore, a well-established functional atlas that covers most of human brain functions need to be further explored.

Two recent rs-fcMRI studies have shown that head motion has significant, systematic effects on functional network measures [28], [70]. In this study, we observed that children and old adults had larger head motion than young adults. To test whether the developmental trajectories of resting-state functional connectivity covary with the motion factor, subjects' mean head motion was regressed from the functional connections that exhibited significant linear or quadratic changes over age (Figure 3). After regression, all the linear and quadratic age effects on functional connectivity were reduced (remaining significant, *p*<0.001) (Figure S4). Thus, head motion can introduce systematic but spurious relationships between age and functional connectivity, but it is not the main variable explaining the human brain functional development. It should be noted that the regression of summary statistics of head motion could not remove all motion-induced artifacts. Carefully controlling the head motion of each subject may be required to refine the results.

### Limitations and future directions

Several limitations should be considered. First, we pooled rs-fcMRI data from two sites to generate a dataset spanning the majority of the lifespan. The two datasets pooled have different voxel size, spatial and temporal signal-to-noise ratios, and total time of acquisition, raising cautions for the interpretation of our findings. However, recent studies have demonstrated the feasibility of sharing and pooling rs-fcMRI data across multiple centers [2], [22]. The general accordance of our findings with previous studies further mitigates this concern. Second, there was limited number of children and old subjects in this study. To avoid overfitting, we only used quadratic trajectories to model nonlinear age effect on resting-state functional connectivity. It is likely that given sufficient number of subjects at different age ranges, more complex developmental trajectories may be detected. Finally, functional disruption of large-scale brain systems has been hypothesized to be responsible for many developmental neuropsychiatric disorders such as Alzheimer's disease [71] and autism [72]. It is interesting to evaluate “brain ages” of patients with neuropsychiatric disorders using the age prediction model learned from normal subjects. Patients may have a larger absolute error between the predicted “brain ages” and chronological ages due to their atypical development of functional brain networks.

## Supporting Information

Figure S1 The LPP embeddings by varying the number of nearest neighbors *k*. Each data point represents one subject. The data points of age from 8 to 79 years are colored from blue to red.
(TIF)

Figure S2 Age-related changes in interregional functional connectivity obtained with unsmoothed data.
(TIF)

Figure S3 Age-related changes in interregional functional connectivity using 160 functional ROIs.
(TIF)

Figure S4 The typically developmental trajectories of resting-state functional connectivity after regression of the mean head motion factor. (A) positive linear change; (B) negative linear change; (C) positive quadratic change; (D) negative quadratic change.
(TIF)

Table S1 The best prediction results of different SVR algorithms using unsmoothed data.
(DOC)

Table S2 The best prediction results of different SVR algorithms using 160 functional ROIs.
(DOC)

Text S1 The algorithmic procedure of LPP.
(DOC)
