# Supplementary figures and images for: Decoding Lifespan Changes of the Human Brain Using Resting-State Functional Connectivity MRI
Source: PLoS One. 2012 Aug 30;7(8):e44530. doi: 10.1371/journal.pone.0044530 (PMC3431403; doi:10.1371/journal.pone.0044530)

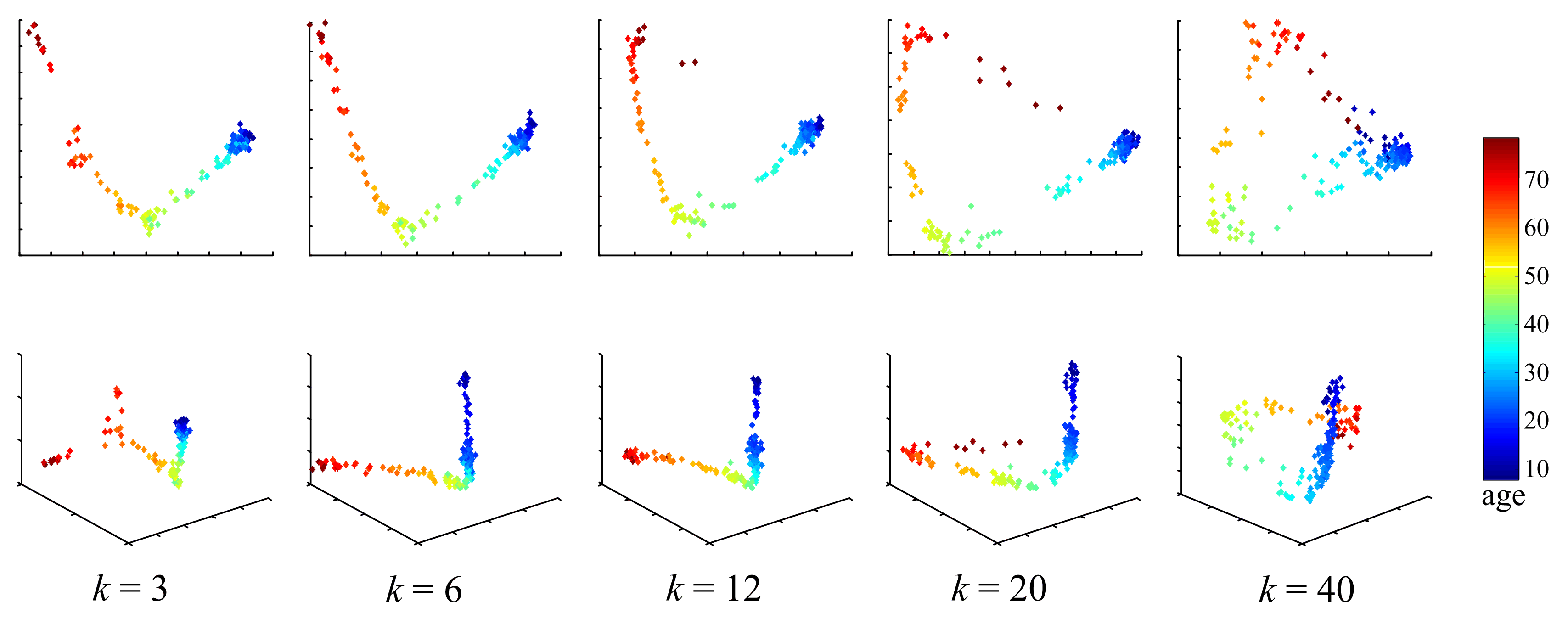

Supplement: Figure S1 — The LPP embeddings by varying the number of nearest neighbors k. Each data point represents one subject. The data points of age from 8 to 79 years are colored from blue to red. (TIF) [file pone.0044530.s001.tif]

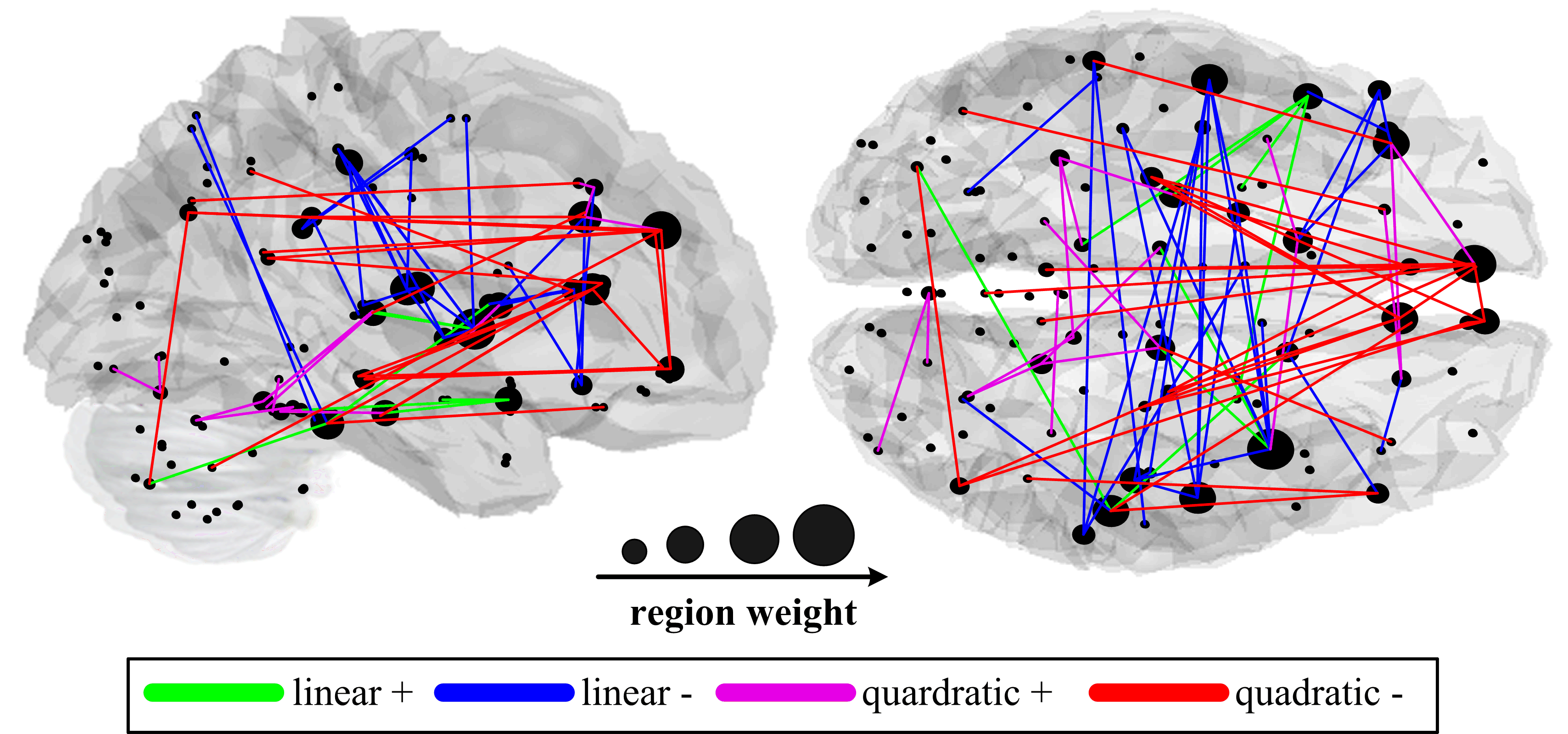

Supplement: Figure S2 — Age-related changes in interregional functional connectivity obtained with unsmoothed data. (TIF) [file pone.0044530.s002.tif]

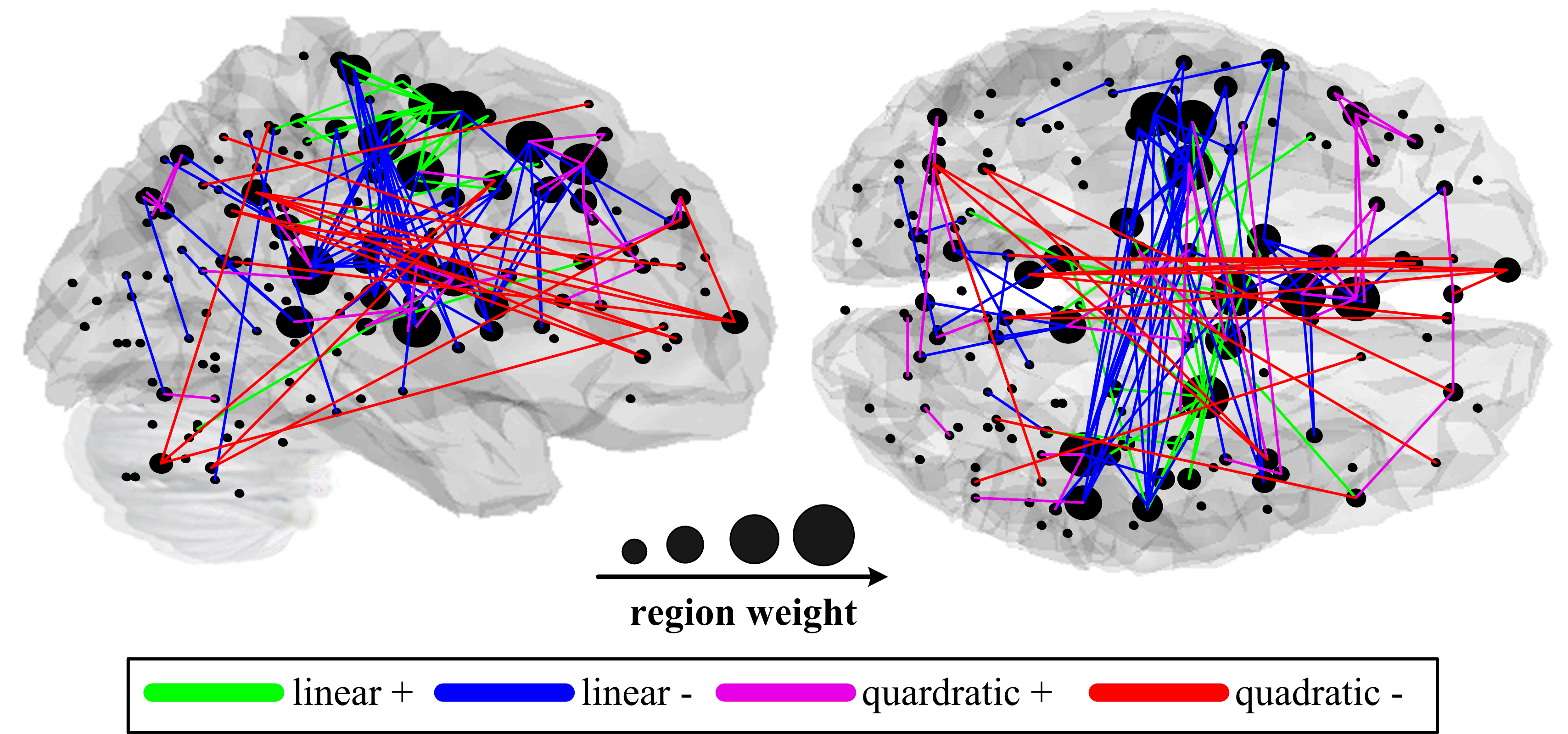

Supplement: Figure S3 — Age-related changes in interregional functional connectivity using 160 functional ROIs. (TIF) [file pone.0044530.s003.tif]

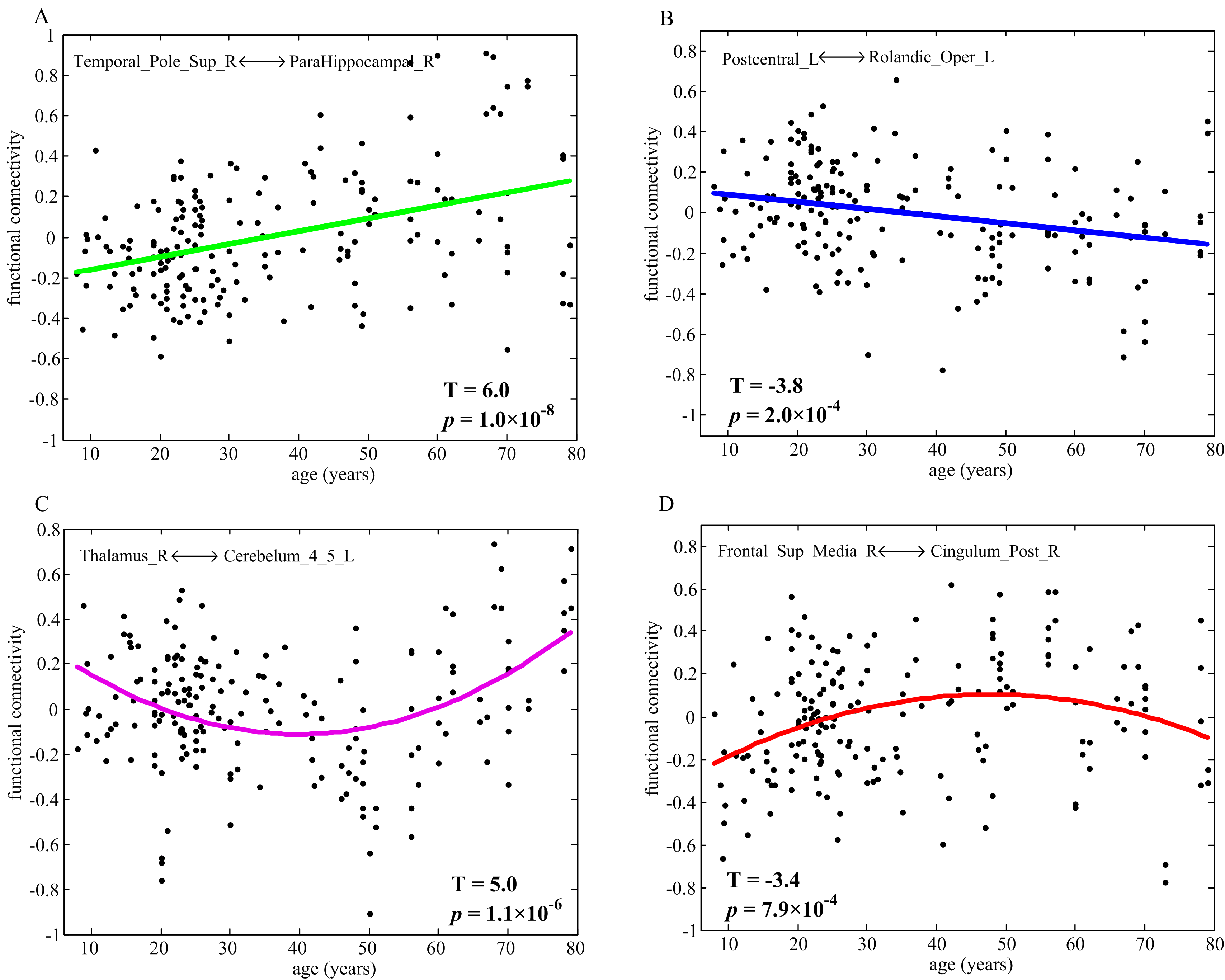

Supplement: Figure S4 — The typically developmental trajectories of resting-state functional connectivity after regression of the mean head motion factor. (A) positive linear change; (B) negative linear change; (C) positive quadratic change; (D) negative quadratic change. (TIF) [file pone.0044530.s004.tif]
