# Supplementary material for: Decoding Lifespan Changes of the Human Brain Using Resting-State Functional Connectivity MRI
Source: PLoS One. 2012 Aug 30;7(8):e44530. doi: 10.1371/journal.pone.0044530 (PMC3431403; doi:10.1371/journal.pone.0044530)
Supplement: Table S2 — The best prediction results of different SVR algorithms using 160 functional ROIs. (DOC) [file pone.0044530.s006.doc]

**Table S2.** The best prediction results of different SVR algorithms using 160 functional ROIs.

| Methods | dimension | MAE | CS(5) | CS (10) | CS(20) |
| --- | --- | --- | --- | --- | --- |
| linear SVR | 6 | 8.4 | 39% | 67% | 93% |
| nonlinear SVR | 4 | 8.3 | 40% | 69% | 92% |
| LASVR (4) | 6 | 8.0 | 42% | 70% | 92% |
| LASVR (8) | 5 | 7.6 | 45% | 74% | 92% |
| LASVR (16) | 10 | 7.7 | 43% | 73% | 93% |
