# Supplementary material for: Decoding Lifespan Changes of the Human Brain Using Resting-State Functional Connectivity MRI
Source: PLoS One. 2012 Aug 30;7(8):e44530. doi: 10.1371/journal.pone.0044530 (PMC3431403; doi:10.1371/journal.pone.0044530)
Supplement: Text S1 — The algorithmic procedure of LPP. (DOC) [file pone.0044530.s007.doc]

**The algorithmic procedure of LPP**

Given a set $\mathbf{x}_{1},...,\mathbf{x}_{N}$ of *N* points in $R^{M}$. The generic problem of LPP is to find a transformation matrix **A** that maps these *N* points to a set of points $\mathbf{y}_{1},...,\mathbf{y}_{k}$ in $R^{m}$ ($m<M$), such that represents where $\mathbf{y}_{i}=\mathbf{A}^{T}\mathbf{x}_{i}$. The algorithmic procedure of LPP is formally stated below:

Step 1. Constructing the adjacency graph. Let G denote a graph with *N* nodes. Nodes *i* and *j* are connected by an edge if *i* is among *k* nearest neighbors of *j* or *j* is among *k* nearest neighbors of *i*.

Step 2. Choosing the weights. Here W is a sparse symmetric *N*×*N* matrix with W_ij_ having the weight of the edge joining nodes *i* and *j*, and 0 if there is no such edge. We put $W_{ij}=\exp\left(-d_{ij}^{2}/t\right)$ if nodes *i* and *j* are connected. The scale parameter $t$ controls the decay of the Gaussian kernel.

Step 3. Eigenmaps. Compute the eigenvectors and eigenvalues for the generalization eigenvector problem:

$\mathbf{XLX}^{T}\mathbf{a}=\lambda\mathbf{XDX}^{T}\mathbf{a}$ (1)

where $\mathbf{D}$ is a diagonal matrix whose entries are column sums of $\mathbf{W}$, $\mathbf{D}_{ii}=\sum_{j} \mathbf{W}_{ji}$. $\mathbf{L}=\mathbf{D}-\mathbf{W}$ is the Laplacian matrix.

Let the column vectors $\mathbf{a}_{0},...,\mathbf{a}_{m-1}$ be the solution of equation (1), ordered according to their eigenvalues, $\lambda_{0},...,\lambda_{m-1}$. Thus, the embedding is as follows:

$\mathbf{x}_{i}\to\mathbf{y}_{i}=\mathbf{A}^{T}\mathbf{x}_{i},\mathbf{A}=\left(\mathbf{a}_{0},...,\mathbf{a}_{m-1}\right)$ (2)

where $\mathbf{y}_{i}$ is a *m*-dimensional vector, and $\mathbf{A}$ is a $M\times m$ matrix.
